# Application of A U-Net for Map-like Segmentation and Classification of Discontinuous Fibrosis Distribution in Gd-EOB-DTPA-Enhanced Liver MRI

**DOI:** 10.3390/diagnostics12081938

**Published:** 2022-08-11

**Authors:** Quirin David Strotzer, Hinrich Winther, Kirsten Utpatel, Alexander Scheiter, Claudia Fellner, Michael Christian Doppler, Kristina Imeen Ringe, Florian Raab, Michael Haimerl, Wibke Uller, Christian Stroszczynski, Lukas Luerken, Niklas Verloh

**Affiliations:** 1 Department of Diagnostic and Interventional Radiology, University Hospital Regensburg, 93053 Regensburg, Germany; 2 Department of Diagnostic and Interventional Radiology, Hannover University Medical Center, 30625 Hannover, Germany; 3 Institute of Pathology, University of Regensburg, 93053 Regensburg, Germany; 4 Department of Diagnostic and Interventional Radiology, Medical Center University of Freiburg, 79106 Freiburg im Breisgau, Germany

**Keywords:** liver fibrosis, cirrhosis, segmentation, Artificial Intelligence, U-Net, convolutional neural network

## Abstract

**Highlights:**

- First application of a U-net for the segmentation and classification of discontinuous liver fibrosis distribution.
- Fully automated, scalable pipeline for data pre-processing, segmentation, and classification.
- The present research could serve as a cornerstone of further applications for non-invasive determination of liver tissue properties, for instance, in planned parenchymal resection.

**Abstract:**

We aimed to evaluate whether U-shaped convolutional neuronal networks can be used to segment liver parenchyma and indicate the degree of liver fibrosis/cirrhosis at the voxel level using contrast-enhanced magnetic resonance imaging. This retrospective study included 112 examinations with histologically determined liver fibrosis/cirrhosis grade (Ishak score) as the ground truth. The T1-weighted volume-interpolated breath-hold examination sequences of native, arterial, late arterial, portal venous, and hepatobiliary phases were semi-automatically segmented and co-registered. The segmentations were assigned the corresponding Ishak score. In a nested cross-validation procedure, five models of a convolutional neural network with U-Net architecture (nnU-Net) were trained, with the dataset being divided into stratified training/validation (*n* = 89/90) and holdout test datasets (*n* = 23/22). The trained models precisely segmented the test data (mean dice similarity coefficient = 0.938) and assigned separate fibrosis scores to each voxel, allowing localization-dependent determination of the degree of fibrosis. The per voxel results were evaluated by the histologically determined fibrosis score. The micro-average area under the receiver operating characteristic curve of this seven-class classification problem (Ishak score 0 to 6) was 0.752 for the test data. The top-three-accuracy-score was 0.750. We conclude that determining fibrosis grade or cirrhosis based on multiphase Gd-EOB-DTPA-enhanced liver MRI seems feasible using a 2D U-Net. Prospective studies with localized biopsies are needed to evaluate the reliability of this model in a clinical setting.

## 1. Introduction

The degree of liver fibrosis and cirrhosis is critical to the prognosis and clinical management of patients with chronic liver disease or patients undergoing liver surgery [1,2]. Taking a liver biopsy is the gold standard in routine clinical practice for measuring liver fibrosis and monitoring response to treatment. The quality of the assessment is directly related to the volume of the sample. Besides interobserver differences, biopsies are prone to misinterpretation in focal disease, absent fibrotic septa, or nodular configurations [3,4].

Moreover, clinical test procedures measure solely the global liver function, which assumes homogeneous distribution. However, liver function seems to be unevenly distributed, especially in patients with cirrhosis, as has been shown, for example, by Tc-99m GSA single-photon emission computed tomography [5]. This limits, for example, the predictive capacity of residual function after partial liver resection, increasing the risk of postoperative liver failure [6].

Ideally, a non-invasive technique would be used to examine not a small section of the liver, but rather, the entire organ. Magnetic resonance imaging (MRI) with hepatocyte-specific contrast agents is routinely used in clinical practice to detect and differentiate hepatic lesions concerning their and the surrounding tissue’s perfusion. In addition to such assessments in the vascular phases, these contrast agents are characterized by an additional hepatobiliary late phase (hepatobiliary phase, HBP) due to their specific enrichment in hepatocytes [7,8,9,10].

This study does not focus on lesion discrimination, but rather, on the liver parenchyma itself. The liver’s signal intensity (SI) is related to perfusion changes during the vascular phases. Active uptake of Gadolinium ethoxybenzyl-diethylenetriaminepentaacetic acid (Gd-EOB-DTPA) into liver cells begins approximately 1 min after contrast administration [11]. Therefore, changes in SI in the hepatobiliary phase are influenced only by the uptake of the contrast agent in hepatocytes [12]. A few studies have shown different enhancement patterns in Gd-EOB-DTPA-enhanced MRI in various stages of liver fibrosis, especially in the HBP. It was observed that, compared to the non-enhanced phase, patients with mild fibrosis or healthy liver parenchyma had a higher increase in the relative SI with time compared to patients with higher levels of fibrosis [13,14]. Therefore, most studies have concentrated on the HBP for assessing liver function. Nevertheless, there also are perfusion changes between liver fibrosis/cirrhosis patients, as shown by color-coded Doppler sonography and 3D whole-liver perfusion MRI [15,16].

With the support of neural networks, segmentation of the liver parenchyma has recently become more accessible, even though it is still a challenge for computational methods due to the wide variation in liver size and shape, especially in the presence of tumors or cirrhosis [17]. Convolutional neural networks with a U-shaped architecture have been proven to be efficacious, not only in organ segmentation, but also in classifications based on these segmentations, for example, in the classification of O(6)-methylguanine-DNA methyltransferase promoter methylation status of brain gliomas based on MRI [18].

This work did not aim to develop a new model architecture. Rather, our objective was to create a non-invasive method to visualize the fibrotic changes of the liver parenchyma and their heterogeneous distribution. For this purpose, we have tested the clinical applicability of the U-Net architecture beyond simple anatomical organ segmentation. Our developed model was able to segment heterogeneous fibrosis distribution and determine the degree of fibrosis/cirrhosis at the voxel level using dynamic Gd-EOB-DTPA-enhanced MRI.

## 2. Materials and Methods

### 2.1. Patients

The local institutional ethics committee approved this retrospective, single-center analysis and waived informed consent. The study was performed following the relevant guidelines and regulations, e.g., the Checklist for Artificial Intelligence in Medical Imaging CLAIM [19] and the Declaration of Helsinki.

**Figure 1:**
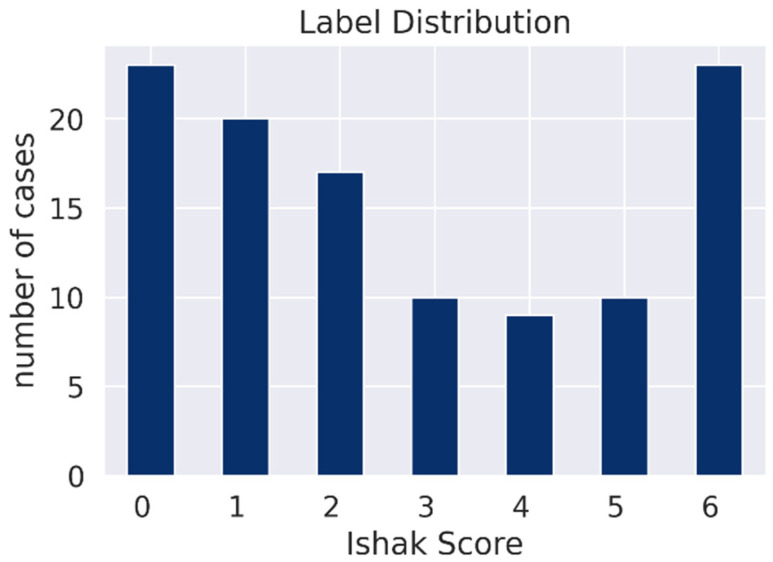
Label distribution. The full dataset’s label distribution (Ishak scores) (*n* = 112).

We included 112 examinations from 112 patients with histologically determined liver fibrosis/cirrhosis grades (Ishak score). Enrolled adult patients had undergone Gd-EOB-DTPA-enhanced MRI and histopathologic examination of the liver between 2013 and 2020 as part of routine clinical practice because of suspected focal liver lesions and tissue inhomogeneities found in ultrasound or to monitor active hepatocellular carcinoma in known liver cirrhosis. No additional examinations were needed. Figure 1 presents the label distribution of the full dataset.

By excluding multiple studies of a single patient, studies of the same patient were not shared between training/validation and test datasets. This also prevented the model from overfitting to specific cases.

Some of the data included in this paper have appeared in previous publications addressing different research questions [20,21,22].

### 2.2. MR Imaging Protocol

All imaging was performed on a clinical whole-body 3T system (Magnetom Skyra, Siemens Healthineers, Erlangen, Germany). For signal reception, a combination of body and spine array coil elements (18-channel body matrix coil, 32-channel spine matrix coil) was used in all examinations. A T1-weighted volume interpolated breath-hold examination (VIBE) sequence with fat suppression (repetition time, 3.09 ms; echo time, 1.17 ms; flip angle 10°; parallel imaging factor, 2; slices, 64; reconstructed voxel size, 1.3 × 1.3 × 3.0 mm^3^; measured voxel size, 1.7 × 1.3 × 4.5 mm^3^) covering the entire liver was acquired in a single breath-hold before contrast injection, during the arterial phase (triggering + 10 s), in the late arterial phase (40 s), in the portal venous phase (75 s), and the hepatobiliary phase (HBP) (20 min). The acquisition time of each VIBE sequence was 14 s, and the delay for the arterial phase was based on triggering in the aorta using an automated CareBolus (Siemens) technique. Gd-EOB-DTPA (Primovist; Bayer Schering Pharma AG, Berlin, Germany) was used as a hepatocytic contrast agent. All patients received a bodyweight adapted Gd-EOB-DTPA (0.025 mmol/kg body weight) administered via bolus injection with a flow rate of 1 mL/s, flushed with 20 mL NaCl. All imaging was consecutively exported from the local Picture Archiving and Communication System (PACS), de-identified, and converted from Digital Imaging and Communications in Medicine (DICOM) to Neuroimaging Informatics Technology Initiative (NIfTI) file format using dcm2niix (version 1.0.20210317, https://github.com/rordenlab/dcm2niix, accessed on 1 April 2021) [23].

### 2.3. Histopathological Examination

Histopathological specimens were obtained from hemi-hepatectomy, (atypical) segment resection, or liver biopsy. The samples were evaluated in a standardized manner according to the institute protocol. Only non-tumorous liver samples were included in this study. Biopsies with tissue lengths less than 15 mm were excluded. Samples were included in the analyses only if more than ten portal veins were visible. All samples were fixed in formalin and embedded in paraffin. Four-micrometer sections were cut vertically and mounted on glass slides. After that, the sections were deparaffinized with xylene and ethanol and stained with hematoxylin-eosin and Elastica van Gieson according to standard protocols. The latter was used to evaluate liver fibrosis. Collagen stained red, and the hepatocytes stained yellow. Two pathologists (K.U. and A.S.), who specialized in liver histopathology, performed the evaluation independently. In case of discrepancies, an additional microscopic analysis was performed collaboratively to reach a joint final assessment. The degree of fibrosis was graded using the Ishak scoring system [24]. The mean time between MRI acquisition and histology was 107.6 days.

### 2.4. Dataset Preparation

Ground truth binary (‘0’ = background, ‘1’ = liver) segmentation of the T1-weighted VIBE sequences was performed as manual proofreading based on pre-segmented MRI by a neural network developed by Winther and colleagues [25]. The manual edge correction was performed using the ITK-SNAP (version 3.8.0, http://www.itksnap.org/pmwiki/pmwiki.php?n=Main.HomePage, accessed on 17 January 2021) open-source software [26]. Malignant (e.g., hepatocellular carcinoma) and benign focal lesions (e.g., simple cysts) were omitted (that is, labeled with the background value ‘0’).

The following steps were performed with Python (version 3.8.12, Python Software Foundation, Wilmington, DE, USA), on a Linux Ubuntu (version 20.04, Canonical Foundation, London, UK) machine with an AMD EPYC 7742 64-Core Processor (AMD, Santa Clara, CA, USA) and four NVIDIA A100-SXM4 40GB GPUs (NVIDIA, Santa Clara, CA, USA).

Because a more precise, localization-dependent determination of the fibrosis score was not available, we approximated by assigning the same score to each voxel of the segmentation mask. For this, the value ‘1’ labeled voxels of the formerly binary segmentations were assigned the corresponding Ishak score (one per patient) as integer values (background, ‘0’; ‘1’, no fibrosis; and ‘7’, cirrhosis). The sequences of native, late arterial, portal venous, and hepatobiliary phases were non-linearly co-registered to the arterial phase by rigid, affine, and symmetric diffeomorphic registration using the advanced normalization tools (ANTs; version 2.3.5, github.com/ANTsX/ANTs, accessed on 5 May 2021) [27] via the Nipype python wrapper [28]. Registration results were visually verified. Scans were then Z-Score normalized and reoriented using FSL Reorient2Std (version 5.0.9; Centre for Functional Magnetic Resonance Imaging of the Brain, Oxford, UK) [29]. 

**Figure 2:**
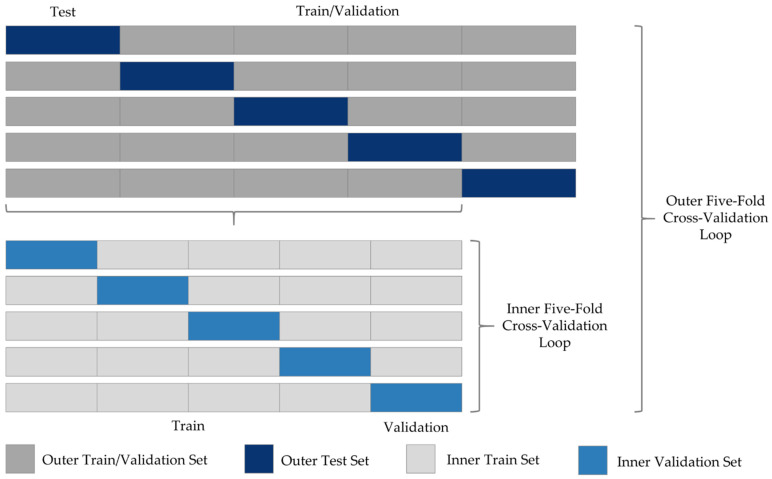
Nested Cross-Validation Procedure. First, the dataset was split into five combinations of 80% training/validation and 20% test cases using stratified sampling to ensure equal label distribution in the whole dataset and all subsets. Second, five models were trained based on the train/validation partitions (outer loop). Per default, nnU-Net employs a second 5-fold cross-validation, for which the train/validation partitions are split into 80% train and 20% test cases (inner loop). Finally, nnU-Net automatically selects the optimal configuration based on the validation partitions. The final model performance is estimated based on the overall prediction scores on the outer loop test sets. Each block represents 20% of the respective data partition.

For the model training, a nested five-fold cross-validation procedure was applied. This method allowed for an evaluation on the entire data set, increasing the validity of the results. This strategy involves an outer and inner loop, both using five-fold cross-validation. Therefore, the dataset cases were first split into five combinations of 80% train/validation (*n* = 89/90) and 20% test cases (*n* = 23/22) using a stratified k-fold. The test cases were retained while the train/validation partitions were used to train five nnU-Net models (outer loop). By default, nnU-Net employs a five-fold cross-validation during model training (inner loop). The dataset split was fixed prior to model training to prevent data leakage. The final model performance was evaluated on the holdout test cases of the outer loop. This procedure ensured that the models were always evaluated on unknown cases. Figure 2 illustrates the nested cross-validation procedure.

### 2.5. Network Training

**Figure 3:**
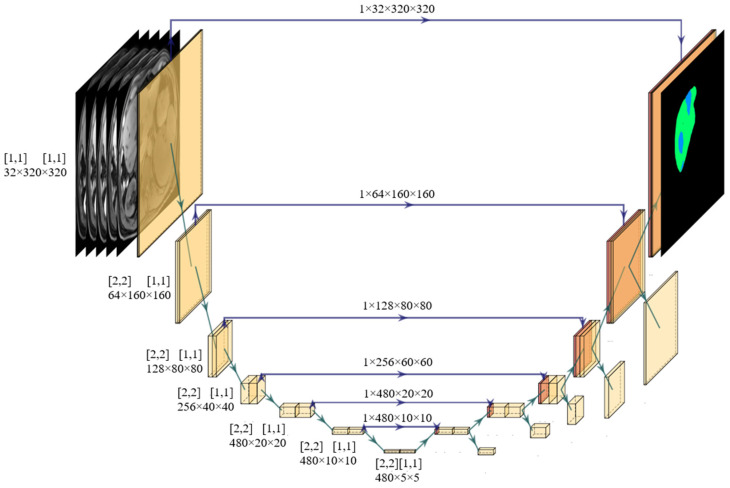
Model Architecture. 2D U-Net Architecture generated by nnU-Net based on the dataset fingerprint [30]. The input consists of the five dynamic T1-weighted MRI sequences. The input patch size is 320 × 320. Yellow planes each represent a sequence composed of plain convolutions (conv), instance normalization (norm), and leaky rectified linear units (ReLU). Conv kernel size is [3, 3] (except for a kernel size of [1, 1] and stride [1, 1] for the segmentation output and auxiliary segmentation output layers). Resolution is reduced after each two of these blocks by strided convolutions (stride is depicted in the right-sided square brackets). Red planes represent transposed convolutions with kernel size [2, 2] and stride [2, 2]. Feature map sizes are displayed for the encoder part (**left**) and mirrored by the decoder (**right**). The illustration in the right upper corner represents an exemplary output segmentation of the model, where the colored pixels depict different Ishak scores and black represents the background value ‘0′. The figure was generated with PlotNeuralNet (version 1.0.0, github.com/HarisIqbal88/PlotNeuralNet, accessed on 21 October 2021).

Five PyTorch-based 2D nnU-Net-models (version 1.6.6, https://github.com/MIC-DKFZ/nnUNet, accessed on 1 July 2021) [30] were trained in a five-fold cross-validation setting with 1000 epochs each (*n*_*train*/*validation*_ = 89/90). All five MRI sequences for each patient were used as input. Stochastic gradient descent was used for optimization with a continuously decreasing learning rate, i.e., the addition of the dice and the cross-entropy form the cost function. See Figure 3 for the full architecture.

### 2.6. Statistical Analysis

Statistical tests were performed using the SciPy library (version 1.7.1, github.com/scipy/scipy, accessed on 3 July 2021) [31]. Kolmogorov–Smirnov tests were used to compare the label distribution in the training and test datasets. Descriptive statistics and two-tailed t-tests compared clinical data. To compare the anatomical segmentations, the resulting masks were binarized and evaluated by their respective Dice similarity coefficient (DICE) and 95% Hausdorff distance metrics (HD95) using the Pymia Python package (version 0.3.1, github.com/rundherum/pymia, accessed on 25 October 2021) [32]. To test the prediction of the Ishak score, for the segmentation masks (being assigned a class label for each voxel), the area under the receiver operating characteristic curve (AUC-ROC), top-three-accuracy score (number of times where the correct label, as determined by the most frequently predicted voxel labels, is among the top three labels predicted), F1-score, precision, and recall as implemented in the scikit-learn library (version 0.24.2, scikit-learn.org/stable/index.html, accessed on 1 April 2021) [33] were used.

## 3. Results

### 3.1. Liver Segmentation

Running inference with the trained nnU-Net models yielded anatomically sound liver segmentation in all test cases. The resulting segmentation maps (assigned integer values ranging from 1–7 corresponding to the Ishak scores 0–6) for every voxel were binarized to quantify anatomical segmentation of the liver volume. For the test cases, the mean DICE was 0.938 (Standard Deviation [SD] = 0.096), and the mean 95% Hausdorff distance (HD95) was 6.015 (SD = 11.912). See Table 1 for results separated by Ishak scores. 

### 3.2. Fibrosis Classification

**Figure 4:**
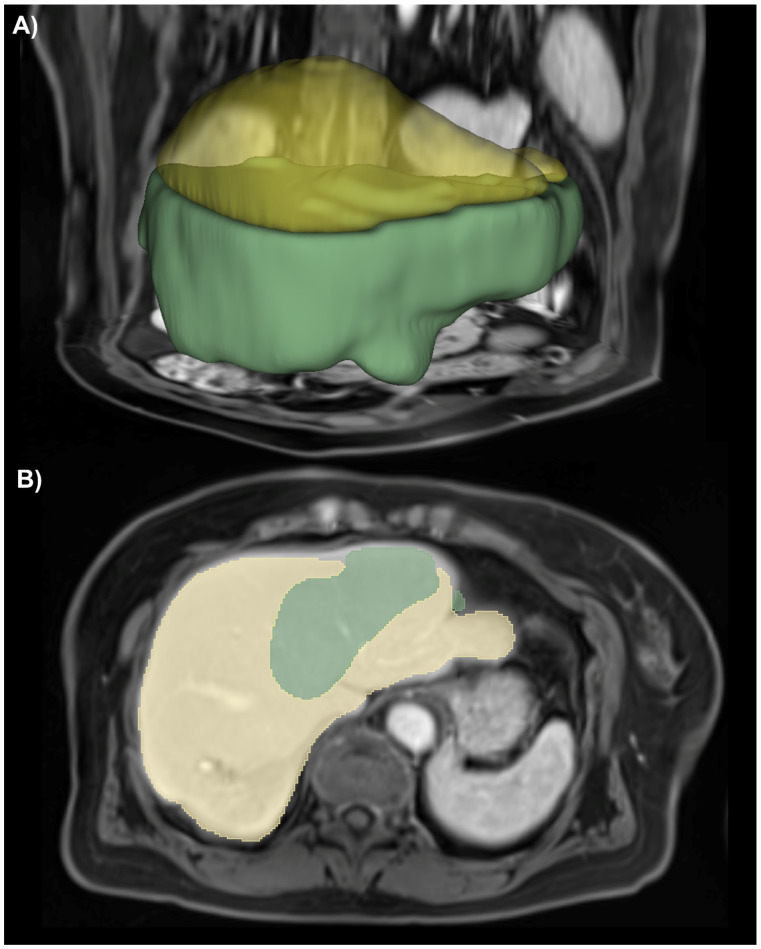
Example segmentation result of fibrosis distribution. (**A**) 3D rendering superimposed on corresponding MRI slices in portal venous phase. (**B**) 2D segmentation mask of the same example. The predictions, in this case, are green = Ishak 0, yellow = Ishak 2.

**Figure 5:**
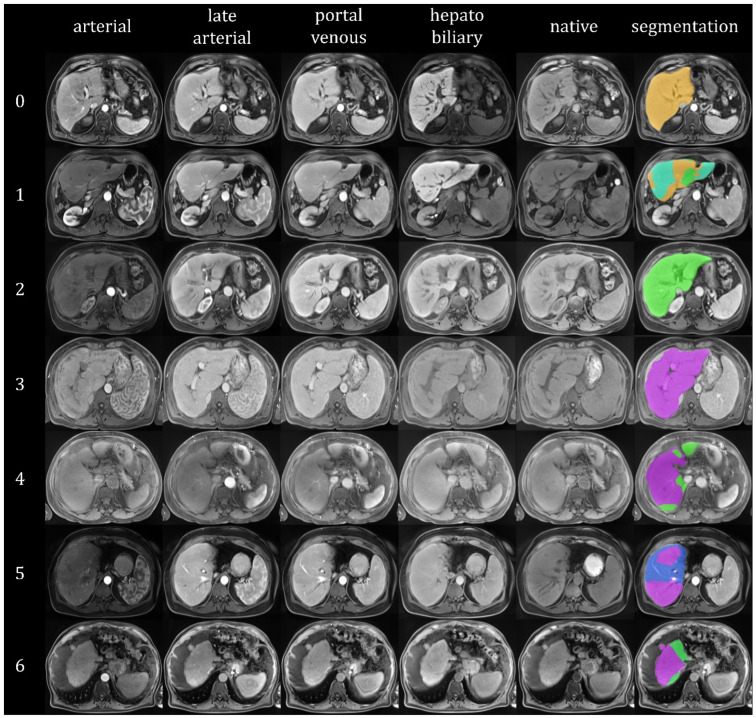
Exemplary dynamic MRI scans and segmentation results. Randomly selected cases. For each Ishak score (as indicated by the numbers on the left), an axial section through the dynamic co-registered and z-score normalized MRI scans in arterial, late arterial, portal venous, hepatobiliary, and native phases as well as the segmentation result superimposed on the portal venous scan is depicted. Colors: orange = Ishak 0, yellow = Ishak 1, green = Ishak 2, light blue = Ishak 3, red = Ishak 4, blue = Ishak 5, purple = Ishak 6. The MRI scans were automatically windowed to display the intensity values between the 0.1 and 99.9 percentiles. It can be seen that the network mainly assigns the values 0, 2, and 6. Value 4 was not assigned, possibly because it was omitted due to its infrequent occurrence during training and optimization.

Since the model assigns an Ishak score to each individual voxel, location-specific predictions of the level of fibrosis are possible. See Figure 4 for an example segmentation. This figure already reveals some limitations of the chosen 2D U-Net architecture. A sharp distinction between the different colored areas can be observed; this seems counterintuitive. Due to the layer-wise prediction of the segmentation, the coherence in the z-direction was compromised, further emphasizing sharp edges in the segmentation. Figure 5 shows the dynamic MRI scans and segmentation results for different histologically determined Ishak scores. 

**Figure 6:**
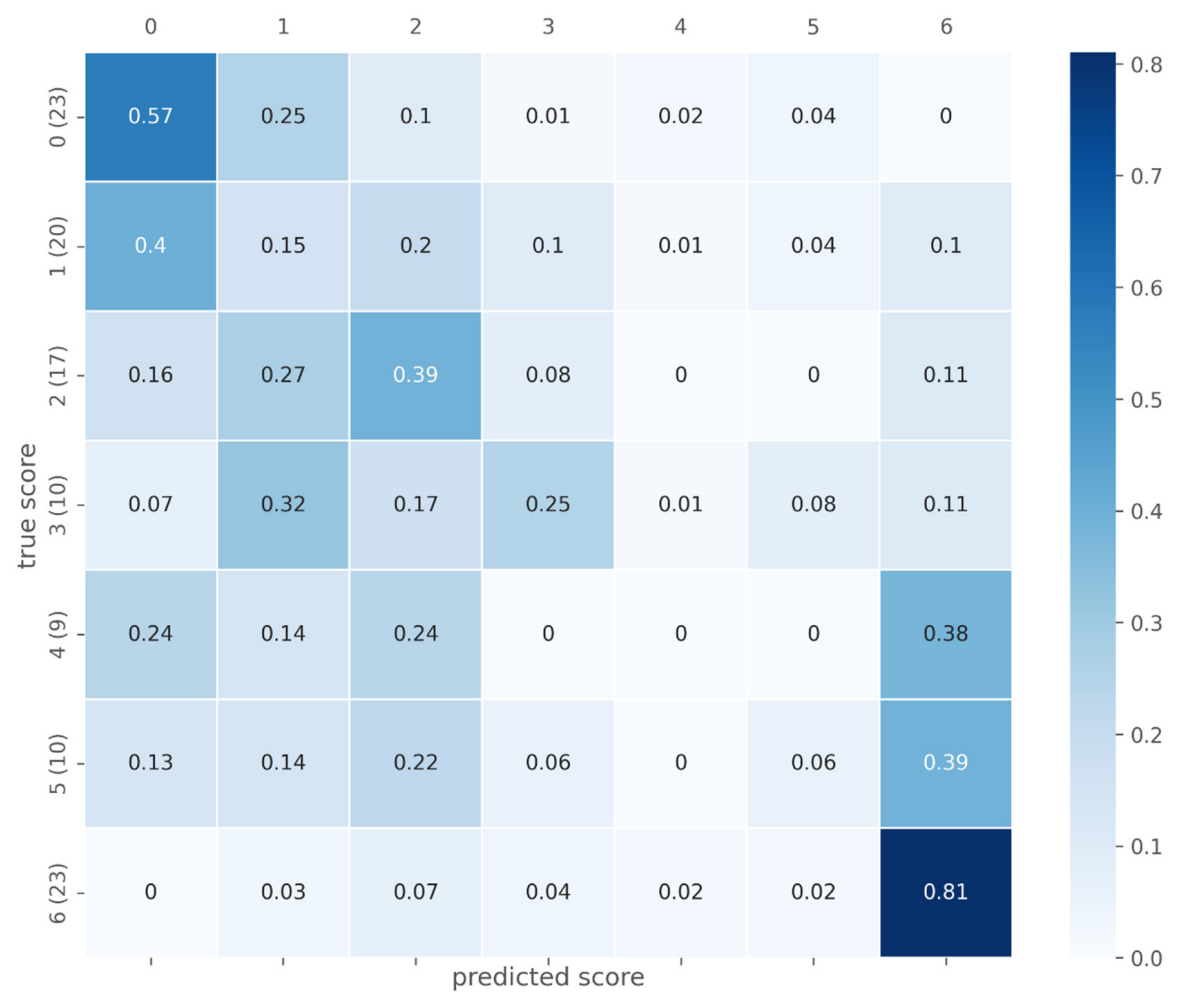
Confusion Matrix. Voxel-level prediction results (proportion) aggregated over all test cases. The number of examples per class is depicted in parentheses.

**Figure 7:**
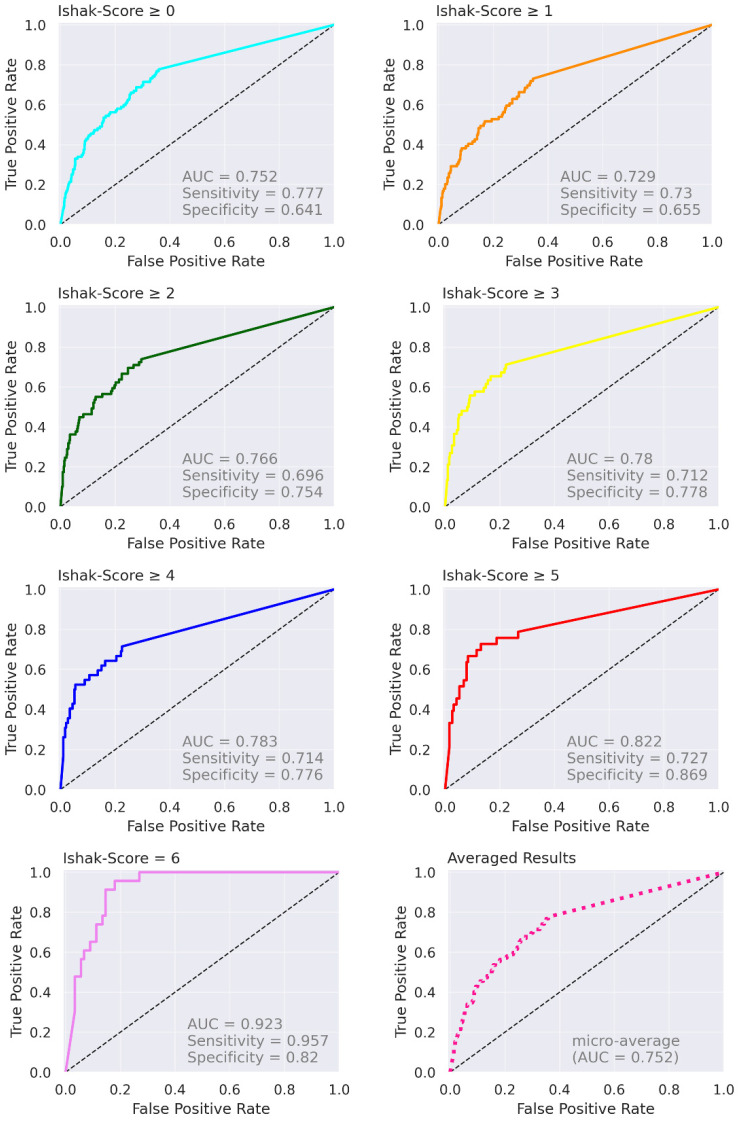
ROC curves. Receiver operating characteristic curves for grouped Ishak scores as well as averaged results (micro-average). Cases were grouped as shown in the plot titles.

The confusion matrix in Figure 6 depicts aggregated voxel-level prediction results for the test data. It can be observed that, especially for Ishak scores 0 and 6, a relatively high number of voxels was classified correctly. In contrast, the classification accuracy for other scores was low (Ishak 1, 2, 3, and 5) or very low (Ishak 4). The receiver operating characteristic curves in Figure 7 show the results for the test data aggregated over all cross-validation folds, separated by the Ishak score.

Considering the test data, the top-three-accuracy score was 0.750, and the micro-average area under the receiver operating characteristic curve (AUC-ROC) was 0.752 for this seven-class classification task. The weighted macro averages of precision, recall, and F1-score were 0.361, 0.429, and 0.383, respectively (with the most prevalent voxel label as the final prediction). According to the Ishak scores, the resulting AUC-ROC values for the test data were Ishak ≥0: 0.752; ≥1: 0.729, ≥2: 0.766; ≥3: 0.780: ≥4: 0.783; ≥5: 0.822; 6: 0.923.

## 4. Discussion

Liver fibrosis and cirrhosis are considered dynamic processes, with the latter being the terminal stage of the former. Cirrhosis is defined as the destruction of the liver parenchyma’s lobules and vessel architecture, as well as nodular regeneration [24]. Tests that predict liver function can lead to customized patient treatment. However, the liver performs several biochemical functions, making functional tests hard to accomplish. Biochemical test methods usually only detect sections of the liver functions. Clinical test procedures only measure global liver function. These methods fail when trying to identify regional dysfunctions when they are compensated for elsewhere. Imaging-based methods offer the advantage of representing global and regional liver function. This study aimed to evaluate the extent to which it is possible to determine the degree of liver fibrosis/cirrhosis at the voxel level in Gd-EOB-DTPA-enhanced MRI of liver using a convolutional neural network with a U-shaped architecture.

### 4.1. Liver Segmentation

As can be observed from Table 1, there was a significant performance drop in the binary segmentation of Ishak 6 labeled cases. This may be explained by the fact that cirrhotic livers vary considerably in size and form. Furthermore, reviewing our cases showed that ascites, which occurs more frequently in advanced liver cirrhosis, poses a problem for our models.

### 4.2. Fibrosis Classification

Several techniques, such as elastography, have been proposed for the non-invasive evaluation of liver fibrosis. MR elastography showed an AUC-ROC of 0.909–0.994 (METAVIR ≥ 2). The corresponding values for ultrasound-based vibration-guided transient elastography were reported to be 0.803–0.914 [34,35,36]. A drawback of these studies is that liver stiffness is only an indirect sign of liver fibrosis; concomitant diseases, such as heart failure also influence liver stiffness. As the uptake of Gd-EOB-DTPA is based on various clinical and biochemical parameters, the HBP is dependent on liver function [13,14,16,37,38,39]. Especially in the case of liver fibrosis/cirrhosis, the uptake of Gd-EOB-DTPA is delayed, accompanied by high diagnostic confidence in both detection and classification of the individual fibrosis grades with an AUC-ROC of ≥0.93 [21,40]. See Table 2 for a comparison of different methods to non-invasively predict the degree of liver fibrosis.

The above studies have in common that histologically determined liver scores, although taken from a small part of the liver in liver biopsies or a segment in liver resection, were used as global liver scores. Samples are often taken from impaired areas, leading to overestimations of the degree of liver fibrosis and potentially imbalanced datasets, as occurred in our case (most cases were labeled as Ishak Scores 0, 1, 2, and 6). The high number of cases of liver cirrhosis might also be explained by the inclusion area of the studies cohort (Upper Palatinate, Southern Germany). The invasive determination of the Ishak score itself also poses problems. Hemi-hepatectomy, (atypical) segment resection, and liver biopsy are in ascending order associated with the risk of sampling error, especially concerning focal liver disease. It is necessary to collect sufficient tissue from multiple locations at best, as the degree of fibrosis may differ between two specimens concurrently taken from the same patient.

Histological determination of the degree of fibrosis itself can also be difficult in some cases. Distinguishing the degree of fibrosis between patients with Ishak scores of 2 (fibrosis expansion in most portal areas ± short fibrosis septa) and 3 (fibrosis expansion in most portal areas with an occasional portal to portal bridging), as well as distinguishing between patients with marked bridging (Ishak 4) only and patients with occasional nodules (incomplete cirrhosis, Ishak 5), leads to discrepancies in histological diagnosis [24,40]. Therefore, in cases of heterogeneous distribution of fibrosis in the liver, sampling results or incorrect sampling during liver biopsy may not represent the entire organ [4,41,42,43]. These limitations also apply to this study. As no voxel-level ground truth was available, the segmentation results could only be evaluated by the histologically determined Ishak score for the entire liver. Also, given the same limitations, the model assigned a separate fibrosis score to each voxel, uncovering a discontinuous fibrosis distribution.

The applied U-Net architecture can accept a theoretically unlimited number of input sequences. This implies that additional MRI scans in order to grade the fibrosis should be incorporated. For example, T2 or diffusion-weighted sequences should be considered in this context. The latter was shown to be on par with MR elastography in grading liver fibrosis without needing the complex mechanical vibration setup [44].

This study has some limitations. First, it only used single-center data from a single scanner, not accounting for the heterogeneity in clinical praxis and thus limiting the model’s generalizability. However, the applied dataset was relatively large and of high quality. nnU-Net was proven to be a versatile framework, achieving state-of-the-art results in various medical imaging segmentation challenges. Second, as shown in Figure 4, applying a 2D U-Net to 3D medical imaging data results in sharp demarcations between the single slices due to the per-slice predictions. This could be reduced by using a 3D architecture. However, in our case, the 2D approach yielded better voxel-wise and overall classification results, which may be attributed to the differences in model architectures (e.g., 2D vs. 3D convolution filters, input sampling).

## 5. Conclusions

In conclusion, determining fibrosis grade or cirrhosis and voxel-wise segmentation, based on multiphase Gd-EOB-DTPA-enhanced liver MRI using a 2D convolutional neural network with U-shaped architecture, seems to be feasible. Our model performed well, especially in cases with no fibrosis (Ishak 0) or cirrhosis (Ishak 6). Moderate fibrosis poses problems for our model, as it does for histology. Prospective studies are needed to clarify the reliability of this model in a clinical setting. This is best done using external validation data and localized biopsies. The model’s fibrosis scoring ability may be optimized by including additional sequences, for example, T2 or diffusion-weighted imaging. The anatomical segmentation and discontinuous fibrosis distribution segmentation could be optimized using a 3D U-Net. We plan to investigate these approaches in the future in a prospective trial with localized biopsies. This approach may also solve the fundamental problem of the missing ground truth at the voxel level.

Finally, this study raises the question of overthinking our understanding of the classification of liver fibrosis/cirrhosis. Is it time to devise a new gold standard?

## Figures and Tables

**Table 1:** Binary segmentation results by Ishak score. This table lists the mean DICE and HD95 for the binarized segmentation results separated by the Ishak score.

	Ishak 0	Ishak 1	Ishak 2	Ishak 3	Ishak 4	Ishak 5	Ishak 6
DICE	0.951	0.935	0.962	0.957	0.951	0.948	0.890
HD95	4.225	5.837	3.015	4.444	4.152	10.004	9.856

**Table 2:** Comparison of different non-invasive methods to predict liver fibrosis. Comparison of different non-invasive methods to predict liver fibrosis taken from the literature as well as results for the proposed model.

	*n*	Compared to	AUC (95% CI)
Magnetic Resonance Elastography			
Bohte et al., 2014 [34]	*n* = 85; F0 (*n* = 3); F1 (*n* = 53); F2 (*n* = 15); F3 (*n* = 8); F4 (*n* = 6)	METAVIR	F ≥ 2, 0.909 (0.840, 0.977); F ≥ 3, 0.928 (0.874, 0.982)
Huwart et al., 2008 [36]	*n* = 96; F0 (*n* = 22); F1 (*n* = 22); F2 (*n* = 19); F3 (*n* = 15); F4 (*n* = 18)	METAVIR	F ≥ 1, 0.962 (0.929, 0.995); F ≥ 2, 0.994 (0.985, 1.0); F ≥ 3, 0.985 (0.968, 1.0); F = 4, 0.985 (0.993, 1.0)
Ultrasound-based Transient Elastography			
Bohte et al., 2014 [34]	*n* = 85; F0 (*n* = 3); F1 (*n* = 53); F2 (*n* = 15); F3 (*n* = 8); F4 (*n* = 6)	METAVIR	F ≥ 2, 0.914 (0.857, 0.972); F ≥ 3, 0.895 (0.816, 0.974)
Huwart et al., 2008 [36]	*n* = 96; F0 (*n* = 22); F1 (*n* = 22); F2 (*n* = 19); F3 (*n* = 15); F4 (*n* = 18)	METAVIR	F ≥ 1, 0.803 (0.701, 0.904); F ≥ 2, 0.837 (0.756, 0.918); F ≥ 3, 0.906 (0.838, 0.975); F = 4, 0.930 (0.877, 0.982)
Uptake of Gd-EOB-DTPA in the HBP			
Verloh et al., 2015 [40]	*n* = 98; 0 (*n* = 17); 1 (*n* = 20); 2 (*n* = 19); 3 (*n* = 5); 4 (*n* = 8); 5 (*n* = 9); 6 (*n* = 20)	Ishak	Ishak ≥ 1, 0.94 (0.90, 1.00); Ishak ≥ 2, 0.93 (0.87, 0.98); Ishak ≥ 4, 0.98 (0.94, 1.00); Ishak = 6, 0.96 (0.92, 0.99)
Haimerl et al., 2017 [21]	*n* = 65; F0 (*n* = 10); F1 (*n* = 14); F2 (*n* = 15); F3 (*n* = 12); F4 (*n* = 14)	METAVIR	F ≥ 1, 1.00 (1.00, 1.00); F ≥ 2, 0.93 (0.89, 0.99); F ≥ 3, 0.98 (0.95, 1.00); F = 4, 0.96 (0.91, 1.00)
2D U-Net			
Proposed (test data)	*n* = 112; 0 (*n* = 23); 1 (*n* = 20); 2 (*n* = 17); 3 (*n* = 10), 4 (*n* = 9), 5 (*n* = 10); 6 (*n* = 23)	Ishak	Ishak ≥ 1, 0.729 (0.59, 0.86); Ishak ≥ 2, 0.766 (0.63, 0.90); Ishak ≥ 4, 0.783 (0.60, 0.97); Ishak = 6, 0.923 (0.85, 1.00)

## Data Availability

All data used for this study are preserved at the Department of Radiology at the University Hospital Regensburg. Data are accessible on-demand as far as allowed by guidelines of the ethics committee of the University Hospital Regensburg. Requests should be addressed to the corresponding author.

## Abbreviations

AUC-ROC	Area Under the Receiver Operating Characteristic Curve
DICE	Dice Similarity Coefficient
Gd-EOB-DTPA	Gadolinium Ethoxybenzyl-Diethylenetriaminepentaacetic Acid
HBP	Hepatobiliary Phase
HD95	95% Hausdorff Distance
MRI	Magnetic Resonance Imaging
SD	Standard Deviation
SI	Signal Intensity
VIBE	Volume-Interpolated Breath-Hold Examination
